# Exploring the Impact of Electric Field and OLi_3_ Decoration on Inorganic Graphenylene SiC for Reversible Hydrogen Storage: A First-Principles Investigation

**DOI:** 10.1021/acsomega.5c12031

**Published:** 2026-02-02

**Authors:** Amit Ramchiary, José A. S. Laranjeira, Paritosh Mondal

**Affiliations:** † Department of Chemistry, 28686 Assam University, Silchar 788011, Assam, India; ‡ Modeling and Molecular Simulation Group, School of Sciences, 28108 São Paulo State University (UNESP), Bauru 17033-360, São Paulo, Brazil

## Abstract

The hydrogen storage capacity of superalkali OLi_3_-decorated inorganic graphenylene SiC (IGP-SiC) has been explored using first-principles calculations employing the GGA-PBE functional. Notably, OLi_3_ is found to be strongly bonded to the IGP-SiC monolayer with a binding energy of −3.89 eV. Thus, a positive charge is developed on the lithium atom of OLi_3_ due to charge redistribution, which enhances its hydrogen adsorption energy. The interaction of H_2_ with OLi_3_@IGP-SiC involves charge polarization as well as orbital and van der Waals interactions. Our calculations reveal a remarkable hydrogen storage capacity of 10.93 wt %, surpassing the DOE-recommended limit of 6.5 wt %, where the hydrogen adsorption energy is found to be in the range of −0.19 to −0.15 eV/H_2_. To assess the thermal stability and reversibility of hydrogen storage exhibited by OLi_3_@IGP-SiC, ab initio molecular dynamics (AIMD) simulations were performed at temperatures of 100, 200, and 300 K. Hydrogen adsorption energy (*E*_H_2__^ad^) of OLi_3_@IGP-SiC can be tuned by applying an electric field. It is noticed that *E*_H_2__^ad^ in OLi_3_@IGP-SiC + 1H_2_ is increased from −0.197 eV/H_2_ (at zero electric field) to −0.657 eV/H_2_ upon application of a +0.055 V/Å electric field. Furthermore, by employing the climbing-image nudged elastic band method, the hydrogen diffusion energy barrier is found to be 0.054 eV. Therefore, the OLi_3_-decorated IGP-SiC monolayer designed in this study may serve as a potential reversible hydrogen storage material.

## Graphical abstract

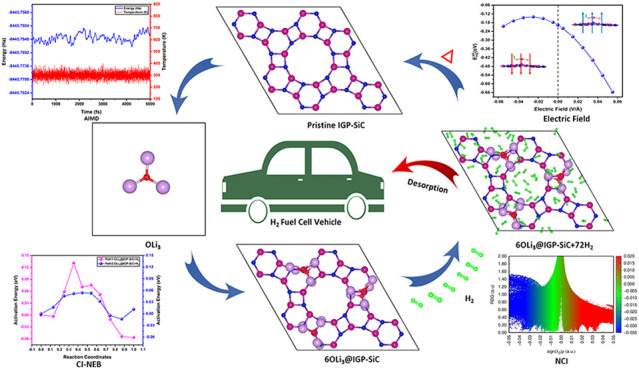

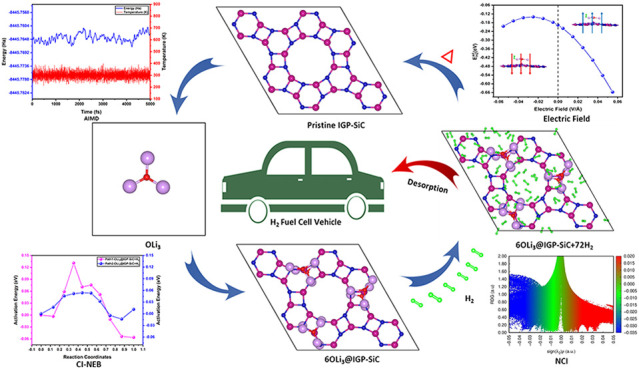

## 1 Introduction

To support the lifestyle of the growing population, the demand for energy is increasing every day. Among various energy sources, fossil fuels are the most widely used, but they are depleting regularly.^1,2^ Additionally, the increased consumption of fossil fuels poses a serious environmental challenge, such as pollution, global warming, etc. Traditionally, most of the energy comes from sources that are not environmentally friendly, posing risks not only to the environment but also to human health. These concerns have given rise to a global push to develop sustainable green energy alternatives. In this scenario, hydrogen emerges as a competitive green energy source compared to fossil fuels because combustion of hydrogen produces only water vapor.^3–6^

Hydrogen is the most abundant and lightest element on Earth and has a gravimetric energy density of 120 MJ/kg, which is almost three times that of gasoline (44 MJ/kg). In contrast, the volumetric energy density of hydrogen is significantly lower than that of gasoline. Since hydrogen is the lightest element, it can be trapped anywhere in the material, exhibiting embrittlement of the storage material. Hence, development of an efficient hydrogen storage method is necessary to support widespread application of hydrogen energy for onboard applications^7–10^ since hydrogen is stored in either physical-based storage or material-based storage. In physical-based storage, hydrogen is stored in the gaseous form as compressed hydrogen and in the liquid form as liquid hydrogen.^11–13^ These physical-based storage methods present safety concerns, loss of energy content, and are expensive and ineffective for onboard practical mobile applications. In contrast, material-based storage or solid-state storage is an alternative method used for hydrogen storage, which is more advantageous in onboard applications, such as in light-duty fuel cell vehicles (FCVs).^14–18^

Solid-state hydrogen storage depends on the adsorbate and adsorbent materials, with either chemisorption of atomic hydrogen or physisorption of molecular hydrogen. Metal hydrides, intermetallic compounds, complex hydrides, graphenes, mxenes, biphenylenes, zeolites, and metal–organic frameworks are some examples of solid-state adsorbent materials.^7,19–21^ To achieve the primary requirements for solid-state hydrogen storage, the U.S. Department of Energy (DOE) has set targets of 6.5 wt % for hydrogen storage capacity, and hydrogen adsorption energy should lie in the range of −0.10 to −0.60 eV/H_2_ under practical conditions of temperature and pressure for reversible hydrogen storage.^22–24^

Particularly, two-dimensional materials show significant potential for achieving high capacity of gravimetric density due to their stability and high surface-to-volume ratios.^25^ Despite their stability and high surface-to-volume ratios, such pristine materials are hardly capable of showing targeted gravimetric density as well as adsorption energy as set by DOE. To overcome these limitations, several modifications have been adopted, such as metal decoration, heteroatom doping, utilization of electric field, or strain and defect engineering.^26–28^ For instance, Zyane et al. reported that Li-decorated GeC_5_ monolayer can achieve a gravimetric hydrogen storage capacity of 7.62 wt % with adsorption energy of −0.22 eV/H_2_.^29^ M. et al. showed that Li-decoration on aza-triphenylene-based covalent organic framework exhibits hydrogen storage capacity of 9.49 wt %, where each of six Li can adsorb up to 5H_2_ having adsorption energy of −0.30 eV/H_2_.^30^ According to Othman et al., hydrogen storage performance of transition-metal-functionalized C_3_N_5_ monolayer is found to be 9.65, 9.48, and 9.32 wt % of H_2_ at 0 K temperature in the cases of Sc, Ti, and V doping, respectively, satisfying the DOE target.^31^ Larangjeira et al. studied Na-decorated TPHE-graphene for promising hydrogen storage applications and reported that each of the Na atoms can adsorb up to 5H_2_, achieving 9.52 wt % with adsorption energy in the range of −0.22 to −0.18 eV.^32^ Similarly, it is also observed that Na-decorated B_7_N_5_ monolayer can adsorb up to 32H_2_, resulting in 7.7 wt % of hydrogen storage.^33^

In contrast, decoration with metal atoms may lead to metal agglomeration due to their high cohesive energy. To circumvent these drawbacks, superalkali-decorated materials may be used for hydrogen storage applications. Superalkali cluster exhibits exceptionally low ionization energy, even lower than that of alkali metals.^34,35^ These materials with low ionization energy can easily donate electrons to enhance the polarization power on the adsorption of hydrogen. Among the superalkalis, OLi_3_ cluster has been first identified by mass spectrometry over solid lithium oxide in 1979.^36^ Since its identification, superalkali OLi_3_ has been found to be an impressive adsorbate for designing materials to enhance hydrogen storage. For instance, Zhang et al. studied OLi_3_-decorated holey graphitic carbon nitride monolayer and reported its gravimetric hydrogen adsorption capacity of 9.45 wt % with average adsorption energy of −0.185 eV per H_2_.^37^ OLi_3_-decorated h-BN nanosheet adsorbs 16H_2_ with adsorption energy of −0.175 eV/H_2_, resulting in hydrogen storage of 9.67 wt %.^38^ You et al. reported that OLi_3_-decorated graphyne and graphdiyne exhibit hydrogen storage capacity of 7.23 and 8.87 wt % with adsorption energy of −0.252 eV/H_2_ and −0.218 eV/H_2_, respectively.^39^ More recently, Laranjeira et al. investigated OLi_3_-decorated irida-graphene and reported that each of OLi_3_ can adsorb up to 12H_2_, reaching the hydrogen storage capacity of 10.0 wt %.^40^

Inorganic graphenylene silicon carbide (IGP-SiC) is a new theoretically predicted two-dimensional semiconductor material with a band gap of 3.22 eV, which contains 4-, 6-, and 12-membered rings. IGP-SiC exhibits high thermal and dynamic stability at approximately 2100 K.^41–43^ Due to its thermal stability, Martins et al. investigated its possible hydrogen storage capability on Li- and Na-decoration and found to be 8.27 and 6.78 wt %, respectively.^44^

Motivated by recent progress on solid-state hydrogen storage, this study investigates the hydrogen storage capacity of an IGP-SiC monolayer using density functional theory (DFT). Initially, the IGP-SiC monolayer has been examined and verified, followed by systematic evaluation of adsorption energy on OLi_3_ decoration at several possible sites within its structure. The binding energy of OLi_3_ on the IGP-SiC is analyzed in detail, and the corresponding thermal stability is checked by ab initio molecular dynamics (AIMD) simulations. Furthermore, the combinations of hydrogen adsorption energy, projected density of states (PDOS), deformation charge density, and noncovalent interactions (NCI) have been investigated to examine the interaction of hydrogen with OLi_3_-decorated IGP-SiC. Moreover, the hydrogen diffusion energy barrier is also examined by the climbing-image nudged elastic band (CI-NEB) method.

## 2 Computational Methods

To explore the hydrogen storage capacity of superalkali OLi_3_-decorated IGP-SiC, the DFT method has been employed using DMol^3^ program package.^45,46^ To analyze electronic and geometrical properties, the generalized gradient-corrected Perdew–Burke–Ernzerhof (PBE/GGA) functional^47,48^ with a double numerical polarization (DNP) basis set has been applied along with Tkatchenko-Scheffler van der Waals (DFT-D) corrections.^49,50^ For core treatment of calculations, DFT semicore pseudopotential (DSPP) is used with a convergence tolerance energy of 1.0 × 10^–5^, maximum force of 0.002 Ha/Å, and maximum displacement of 0.005 Å. Global orbital cutoff of 4.5 Å is set in all simulations, and 5 × 5 × 1 and 10 × 10 × 1 *k*-points grids are employed to sample the Brillouin zone for geometry relaxation and electronic property calculations, respectively.^51^ Ab initio molecular dynamics (AIMD) simulations have been performed using the same DFT code to compute the thermal structural integrity and reversible nature of hydrogen storage with *NVT* ensemble and Nose–Hoover Chain thermostat with a time step of 1 fs at three temperatures for 5 ps simulations.

Quantum ESPRESSO package has been employed to analyze hydrogen diffusion barrier using the CI-NEB method. To treat the core electrons, ultrasoft pseudopotentials^52–54^ have been used along with an energy cutoff of 45 Ry and a charge density of 450 Ry. The integration of the Brillouin zone is performed at the Γ-point within the Monkhorst–Pack Scheme.^51^ The Multiwfn code is further deployed to analyze the NCI index^55^ in the studied materials.

The average binding energy of OLi_3_ on different adsorption sites of IGP-SiC is calculated using the equation^56^

1 $$E_{b}=\left(\frac{E_{mOLi_{3}@IGP‐SiC}-E_{IGP‐SiC}-mE_{OLi_{3}}}{m}\right)$$

where *m* is the number of superalkali OLi_3_ clusters, and $E_{mOLi_{3}@IGP‐SiC}$ , *E*_IGP‑SiC_, and $E_{OLi_{3}}$ are the total energy of $E_{mOLi_{3}@IGP‐SiC}$ , pristine IGP-SiC, and isolated OLi_3_ cluster, respectively.

The adsorption energy per H_2_ ( $E_{H_{2}}^{ad}$ ) on OLi_3_-decorated IGP-SiC has been calculated using the expression^57^

2 $$E_{H_{2}}^{ad}=\left(\frac{E_{mOLi_{3}@IGP‐SiC+nH_{2}}-E_{mOLi_{3}@IGP‐SiC}-nE_{H_{2}}}{n}\right)$$

where *m* and *n* represent the number of OLi_3_ clusters and H_2_, respectively. $E_{mOLi_{3}@IGP‐SiC+nH_{2}}$ , $E_{mOLi_{3}@IGP‐SiC}$ , and *E*_H_2__ represent the total energy of hydrogen adsorbed OLi_3_@IGP-SiC, OLi_3_@IGP-SiC monolayer, and isolated H_2_, respectively.

The hydrogen storage gravimetric capacity (H_2_, wt %) of the OLi_3_-decorated IGP-SiC monolayer is calculated using the following equation

3 $$H_{2}\;\left(wt\;\%\right)=\left(\frac{nM_{H_{2}}}{nM_{H_{2}}+M_{mOLi_{3}@IGP‐SiC}}\right)\times 100$$

where *nM*_H_2__ and $M_{mOLi_{3}@IGP‐SiC}$ refer to the mass of the *n*H_2_ molecule adsorbed and *m*OLi_3_-decorated IGP-SiC system, respectively.

The desorption temperature (*T*_D_) is calculated using the van’t Hoff equation

4 $$T_{D}=\frac{E_{H_{2}}^{ad}}{\kappa_{B}}{\left(\frac{\Delta S}{R}-\ln\;P\right)}^{-1}$$

where Δ*S*, *R*, and *P* represent the change in entropy from gas to liquid phase (Δ*S* = 75.44 J K^–1^ mol^–1^), the universal gas constant, and atmospheric pressure, respectively.

The adsorption–desorption cycle of adsorbed hydrogen on OLi_3_@IGP under practical conditions of temperature and pressure is calculated using statistical thermodynamics. The hydrogen occupation number (*N*) is calculated using the mathematical formula given below

5 $$N=\frac{\sum_{n=0}^{n_{\max}}ng_{n}\;\exp\left[\frac{n\left(\mu -E_{ad}\right)}{\kappa_{B}T}\right]}{\sum_{n=0}^{n_{\max}}g_{n}\exp\left[\frac{n\left(\mu -E_{ad}\right)}{\kappa_{B}T}\right]}$$

where *n*_max_ is the highest number of adsorbed hydrogens per OLi_3_@IGP-SiC at 0 K. While *n*, *k*_B_, *g*_n,_ and *E*_ad_ represent the number of adsorbed H_2_ per site, the Boltzmann constant, the degeneracy (*g*_n_ = *1*), and the hydrogen adsorption energy, respectively.

However, μ_H_2__(*T*,*P*) is the chemical potential of H_2_ at temperature (*T*) and pressure (*P*), which is defined as

6 $$\mu_{H_{2}}\left(T,P\right)=H_{\left(T\right)}^{0}-TS_{\left(T\right)}^{0}+\kappa_{B}T\;\ln\left(\frac{P}{P_{0}}\right)$$

where *H*_(*T*__)_^°^ and *S*_(*T*__)_^°^ represent the enthalpy and entropy of hydrogen at temperature *T*, which are taken from the experimental database.^58^ *H*_(0)_^°^ and *P*_0_ are the enthalpy at 0 K (equal to zero) and the reference pressure (equal to 1 bar), respectively.

## 3 Results and Discussion

### 3.1 Structure of Superalkali OLi_3_-Anchored IGP-SiC

The unit cell of inorganic graphenylene silicon carbide (IGP-SiC), having a hexagonal lattice, belongs to the space group *P*6/*m* (no. 175), consisting of six carbon and six silicon atoms. The lattice parameters of the optimized unit cell of IGP-SiC are *a* = *b* = 8.44 Å, as shown in Figure S1, which is in agreement with available literature reports.^43^ However, in our current study, a 2 × 2 × 1 supercell of the IGP-SiC unit cell is chosen for investigation. As shown in Figure 1a, the relaxed structure exhibits lattice parameters of *a* = *b* = 16.9 Å with each of 24 carbon and silicon atoms. There are three distinct Si–C bonds, labeled as b1, b2, and b3, as illustrated in Figure 1a and measured to be 1.84, 1.75, and 1.80 Å, respectively. However, six different bond angles are measured to be as θ1 = 86.18°, θ2 = 93.82°, θ3 = 118.45°, θ4 = 121.41°, θ5 = 155.59°, and θ6 = 144.82°. In contrast, the relaxed structure of the superalkali OLi_3_ cluster has trigonal planar geometry and is depicted in Figure 1b. DFT-evaluated O–Li bond length and Li–O–Li bond angle is found to be 1.7 Å and 119.99°, respectively, closely matching with previous results.^59^

**Figure 1:**
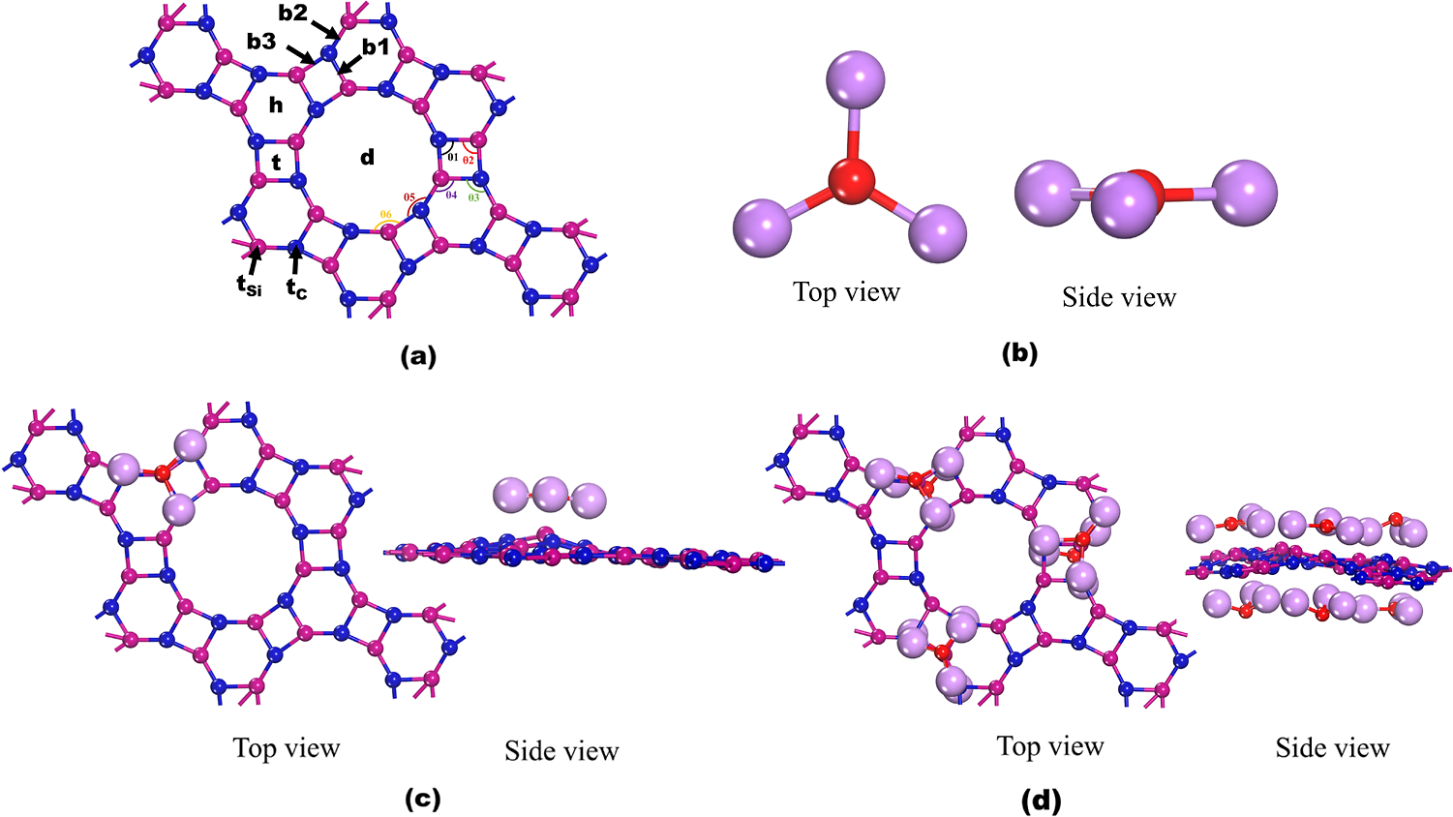
DFT-evaluated optimized geometries of (a) IGP-SiC, (b) OLi_3_, (c) OLi_3_@IGP-SiC, and (d) 6OLi_3_@IGP-SiC. Blue, pink, red, and light purple colors denote carbon, silicon, oxygen, and lithium atoms.

In this study, superalkali OLi_3_ is allowed to adsorb on different sites of IGP-SiC, as shown in Figure 1a. The adsorption sites are depicted as h (above the hexagonal ring), t (above the tetragonal ring), d (above the dodecagonal ring), t_C_ (above the carbon atom), and t_Si_ (above the silicon atom). Moreover, bridge positions are labeled as b1 (above the bond between hexagonal and tetragonal), b2 (above the bond between hexagonal and dodecagonal), and b3 (above the bond between tetragonal and hexagonal). In each system, the OLi_3_ cluster is allowed to interact from 2 Å above the IGP-SiC monolayer, and then the obtained geometry is relaxed. It is observed that, except for the h and d sites, in all other decoration sites, OLi_3_ cluster is seen to have migrated toward the nearest t_Si_ sites, as illustrated in Figure S2. However, decoration with multiple OLi_3_ clusters on h and d sites is also seen to have migrated toward the silicon atom, as represented in Figure S3. This is due to the interaction of the electronegative oxygen atom with the electropositive silicon atom, which is even more electropositive than the carbon atom. After analyzing the migration and deformation of the geometry, it is found that the optimized OLi_3_@IGP-SiC geometry, as shown in Figure 1c, is the most stable. The binding energy (*E*_b_), calculated using eq 1, illustrates the binding strength of OLi_3_ on the IGP-SiC monolayer. Binding energy of the system is found to be −3.86 eV/OLi_3_. Notably, this binding energy is much higher than the cohesive energy of the Li atoms, circumventing agglomeration of lithium atoms leading to the formation of stable OLi_3_@IGP-SiC. Therefore, the stable OLi_3_@IGP-SiC cluster has been chosen for further study of hydrogen storage. The top and side view of OLi_3_@IGP-SiC is shown in Figure 1c. Distance between the oxygen atom of OLi_3_ and the silicon atom of IGP-SiC is measured to be 1.74 Å. A significant variation of geometrical parameters is noticed in IGP-SiC on interaction with OLi_3_. The Si–C bond lengths in b1, b2, and b3 change to 1.85, 1.76, and 1.86 Å, respectively. Moreover, the O–Li bond length and the Li–O–Li bond angle are calculated to be 1.91 Å and 119.93°, respectively. Observed geometrical changes attested the formation of the OLi_3_@IGP-SiC monolayer. This study is further extended to form 6OLi_3_@IGP-SiC on decoration with three OLi_3_ on either side of the IGP-SiC monolayer, maintaining the optimum distance to avoid agglomeration of OLi_3_ clusters, and the optimized geometry is given in Figure 1d. Binding energy of OLi_3_ on the IGP-SiC monolayer is estimated to be −3.43 eV/OLi_3_. Furthermore, the work function, which is the energy required to remove an electron from the surface of pristine IGP-SiC and OLi_3_@IGP-SiC, is measured to be 5.17 and 4.68 eV, respectively (Figure 2a). This reduction in the work function reveals a stronger interaction between the OLi_3_ cluster and the IGP-SiC monolayer. Additionally, electronic band structures have been calculated at the GGA/PBE level, as illustrated in Figure S4. Pristine IGP-SiC exhibits an underestimated band gap of 2.17 eV, however, the OLi_3_@IGP-SiC has band gap of 1.91 eV. This reduction in band gap indicates the n-type doping behavior due to the charge donation from OLi_3_ to the IGP-SiC monolayer, which in turn enhances its conductivity property, enabling faster electron transfer, leading to the design of better materials for hydrogen storage applications.

**Figure 2:**
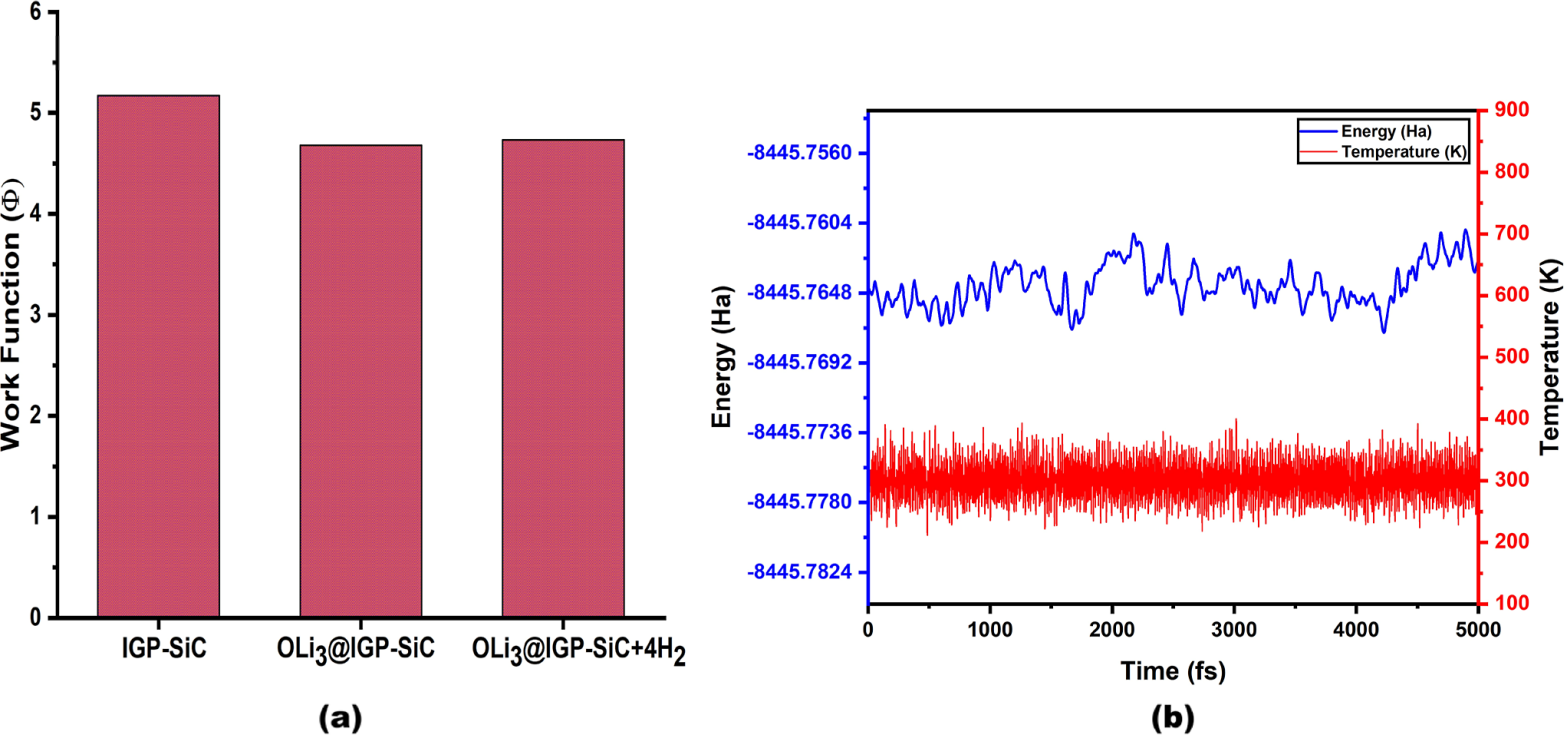
DFT-evaluated (a) work function for pristine IGP-SiC, OLi_3_@IGP-SiC, and OLi_3_@IGP + 4H_2_ and (b) ab initio molecular dynamics (AIMD) simulation results for 6OLi_3_@IGP-SiC, computed at 300 K to assess thermal stability.

To check the thermal stability of 6OLi_3_@IGP-SiC, AIMD simulation has been carried out using an *NVT* ensemble at 300 K for 5 ps simulation. The simulation indicates stability of 6OLi_3_@IGP-SiC, which is evident from the minor fluctuations in the total energy as shown in Figure 2b. Therefore, the calculated binding energy and AIMD simulation support the hydrogen storage ability of OLi_3_@IGP-SiC.

### 3.2 Hydrogen Storage Properties of Superalkali OLi_3_ Anchored IGP-SiC

To investigate the hydrogen storage performance of OLi_3_@IGP-SiC and 6OLi_3_@IGP-SiC, a hydrogen molecule is loaded stepwise, one by one, sequentially to reach the saturation point of hydrogen adsorption. First, the adsorption of hydrogen on OLi_3_@IGP-SiC has been investigated to analyze the saturation of hydrogen adsorption per OLi_3_. To begin with, 4H_2_ are allowed to adsorb on OLi_3_@IGP-SiC (OLi_3_@IGP-SiC + 4H_2_) and then relax the geometry, which is illustrated in Figure 3a. The hydrogen adsorption energy ( $E_{H_{2}}^{ad}$ ) is estimated to be −0.19 eV using eq 2. It is observed that the H–H bond length of adsorbed H_2_ is elongated to 0.760 Å from 0.751 Å. On the other hand, the Li–H_2_ distance is measured to be 2.13 Å (Table 1). In the next steps, $E_{H_{2}}^{ad}$ of OLi_3_@IGP-SiC + 8H_2_ (Figure 3b) and OLi_3_@IGP-SiC + 12H_2_ (Figure 3c) has been computed to be −0.17 eV and −0.15 eV, respectively, at the PBE/DNP level. H–H and Li–H_2_ distances in the case of optimized OLi_3_@IGP-SiC + 12H_2_ were measured to be 0.757 and 3.55 Å, respectively.

**Figure 3:**
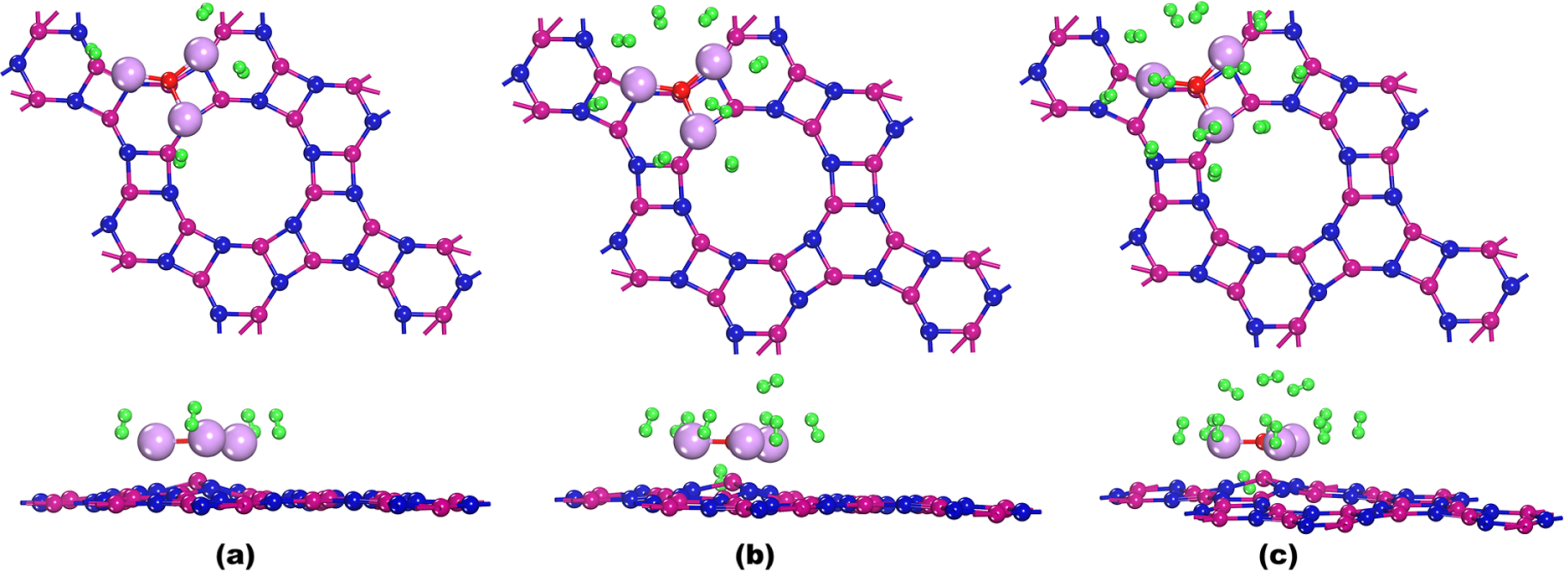
Top and side views of the relaxed structures of H_2_ adsorption systems: (a) OLi_3_@IGP-SiC + 4H_2_, (b) OLi_3_@IGP-SiC + 8H_2_, (c) OLi_3_@IGP-SiC + 12H_2_. Blue, pink, red, light purple, and green colors denote carbon, silicon, oxygen, lithium, and hydrogen atoms.

**Table 1:** Adsorption of Hydrogen Molecule on OLi_3_@IGP-SiC, Including H_2_ Adsorption Energy per H_2_ Molecule (*E*_H_2__^ad^), Average H–H Bond Distance (Å), Average Li–H_2_ (Å), Desorption Temperature (*T*_D_), and Hydrogen Gravimetric Capacity (wt %)

system	*E*_H_2__^ad^ (eV/H_2_)	bond distance	bond distance	*T*_D_ (K)	gravimetric capacity (wt %)
		H–H (Å)	Li–H_2_ (Å)		
OLi_3_@IGP-SiC + 4H_2_	–0.19	0.757–0.760	2.05–2.13	243	0.80
OLi_3_@IGP-SiC + 8H_2_	–0.17	0.751–0.758	2.09–3.46	217	1.59
OLi_3_@IGP-SiC + 12H_2_	–0.15	0.751–0.757	2.17–3.55	192	2.36
6OLi_3_@IGP-SiC + 24H_2_	–0.19	0.755–0.769	1.95–3.36	243	3.93
6OLi_3_@IGP-SiC + 48H_2_	–0.18	0.751–0.766	1.92–3.67	230	7.56
6OLi_3_@IGP-SiC + 72H_2_	–0.15	0.751–0.767	1.95–4.44	192	10.93

Similarly, the hydrogen adsorption capacity of 6OLi_3_@IGP-SiC per OLi_3_ was investigated in detail by using the quantum chemical method. DFT-evaluated relaxed geometries and their significant geometrical parameters of hydrogen-adsorbed 6OLi_3_@IGP-SiC have been depicted in Figure 4 and Table 1, respectively. As shown in Figure 4a, the hydrogen adsorption energy of 24H_2_ adsorbed on 6OLi_3_@IGP-SiC (4H_2_ per OLi_3_) is calculated to be −0.19 eV, where H–H and Li–H_2_ distances are measured to be 0.769 and 3.36 Å, respectively. Moreover, $E_{H_{2}}^{ad}$ of 6OLi_3_@IGP-SiC + 48H_2_ (Figure 4b) and 6OLi_3_@IGP-SiC + 72H_2_ (Figure 4c) is found to be −0.18 eV and −0.15 eV, respectively. This reduction of adsorption energy with an increased number of adsorbed H_2_ is due to the weak interaction between hydrogen and IGP-SiC. After adsorption of 12H_2_ per OLi_3_ (OLi_3_@IGP-SiC + 12H_2_ and 6OLi_3_@IGP-SiC + 72H_2_ are achieved), the saturation point of hydrogen adsorption, which is confirmed from the calculated H–H bond length (0.751 Å), is the same as that of isolated H_2_.

**Figure 4:**
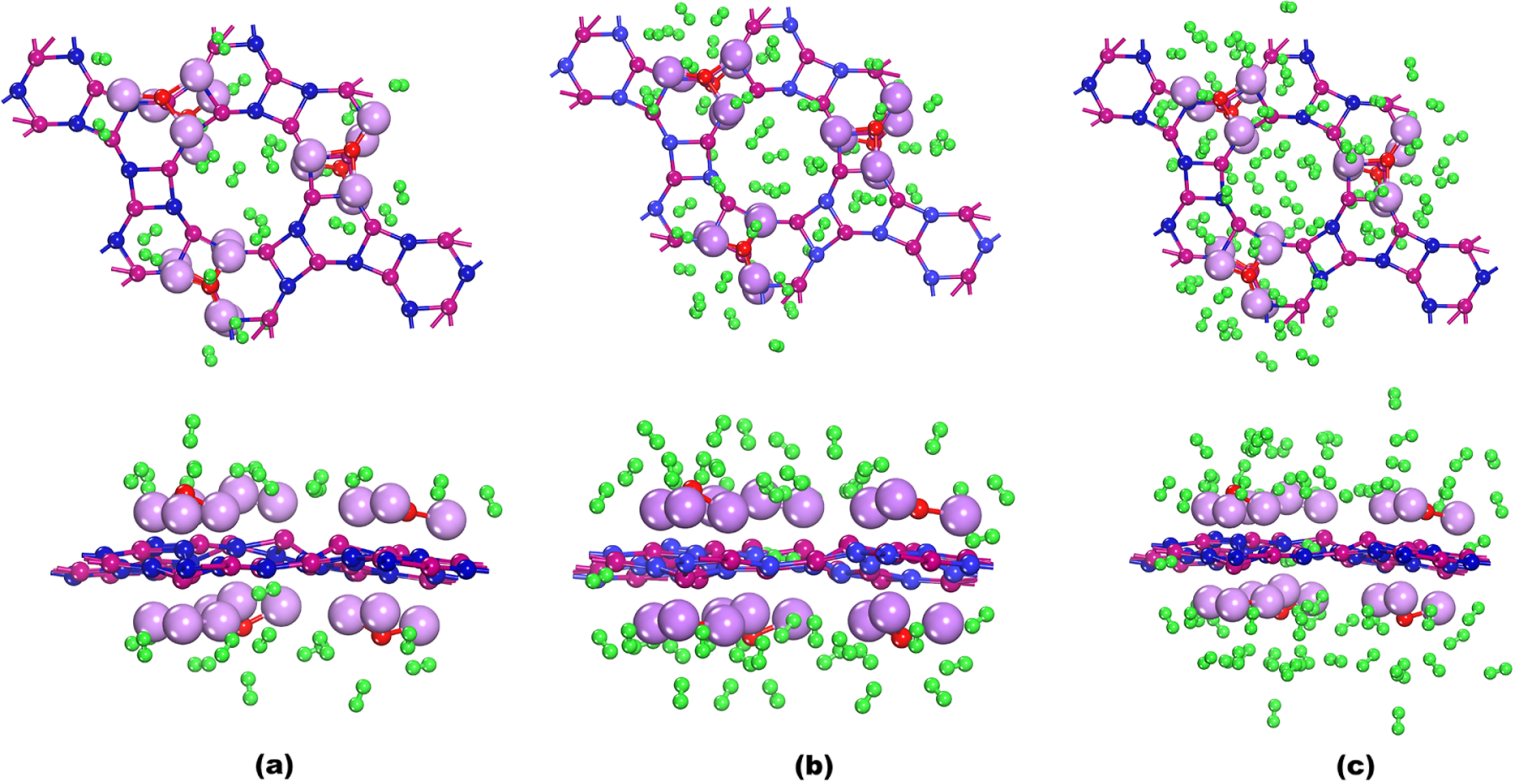
Top and side views of the relaxed geometries of H_2_ adsorbed (a) 6OLi_3_@IGP-SiC + 24H_2_, (b) 6OLi_3_@IGP-SiC + 48H_2_, (c) 6OLi_3_@IGP-SiC + 72H_2_.

The hydrogen storage capacity of OLi_3_-decorated IGP-SiC has been calculated by eq 3. The adsorption energy ( $E_{H_{2}}^{ad}$ ) and gravimetric storage capacity are provided in Table 1. It is observed from Table 1 that OLi_3_@IGP-SiC + 12H_2_ reaches a hydrogen storage capacity of 2.36 wt %. On the other hand, 6OLi_3_@IGP-SiC + 72H_2_ exhibits the highest gravimetric capacity of 10.93 wt %, surpassing the proposed DOE target of 6.5 wt % by 2025. Furthermore, a comparison table of gravimetric hydrogen storage capacities for different storage materials based on the literature has been prepared and presented in Table 2. Table 2 shows that the material designed in this study (OLi_3_@IGP-SiC) outperformed the hydrogen storage capacity of several materials mentioned in the literature. Thus, OLi_3_@IGP-SiC satisfies the criteria of gravimetric density as well as adsorption energy per the DOE target. Therefore, OLi_3_@IGP-SiC may be utilized as a promising material for hydrogen storage.

**Table 2:** Comparison of Different Hydrogen Storage Materials in Terms of Adsorption Energy per H_2_ ( $E_{ad}^{H_{2}}$ ), Gravimetric Weight Percentage (wt %), and Desorption Temperature (*T*_D_)

system	$E_{ad}^{H_{2}}$ (eV/H_2_)	wt %	*T*_D_ (K)	reference
**OLi**_**3**_ **@IGP-SiC**	**–0.17**	**10.93**	**192**	**present work**
Li@GeC_5_ monolayer	–0.22	7.62	230	29
Li@AzaCOF	–0.30	9.49	330	30
Sc/Ti/V@C_3_N_5_	–0.27/–0.29/–0.24	9.65/9.48/9.32	--	31
Li@TPHE-graphene	–0.22 to −0.18	9.52	243.47	32
Na@B_7_N_5_	–0.20	7.7	257	33
NLi_4_@ γ-graphyne	–0.167	6.78	213	35
OLi_3_@CN monolayer	–0.185	9.45	137	37
OLi_3_@h-BN nanosheet	–0.175	9.67	234	38
OLi_3_@graphyne/graphdiyne	–0.252/–0.218	7.23/8.87	424/367	39
OLi_3_@irida-graphene	–0.19	10.00	238	40
Li/Na@IGP-SiC	–0.140/–0.099	8.27/6.78	191/148	44

### 3.3 Mechanism of H_2_ Adsorption on OLi_3_@IGP-SiC

Adsorption of hydrogen molecules on OLi_3_@IGP-SiC can be understood well by analyzing the deformation charge density, Hirshfeld charge analysis, PDOS plots, and noncovalent interaction (NCI) analysis. Figure 5a–d shows the deformation charge density, which reveals the depletion and accumulation of charges in different regions of the material. The red color signifies the charge accumulation, while the blue color represents the charge depletion. Figure 5a represents the deformation charge density of pristine IGP-SiC, whereas the deformation charge density of OLi_3_@IGP-SiC is presented in Figure 5b. In the case of OLi_3_@IGP-SiC, the red color on the oxygen atom of OLi_3_ indicates accumulation of electronic charge, while depletion of electronic charge is noticed on lithium atoms. Depletion of the electronic charge creates positive charge on lithium atoms, thereby facilitating polarized adsorption of H_2_. Moreover, the deformation charge density of OLi_3_@IGP-SiC + 4H_2_ and OLi_3_@IGP-SiC + 12H_2_ is illustrated in Figure 5c,d. The charge depletion and charge accumulation can be observed on the adsorbed hydrogen atoms. This defines the charge redistribution between adsorbed hydrogen atoms and OLi_3_@IGP-SiC.

**Figure 5:**
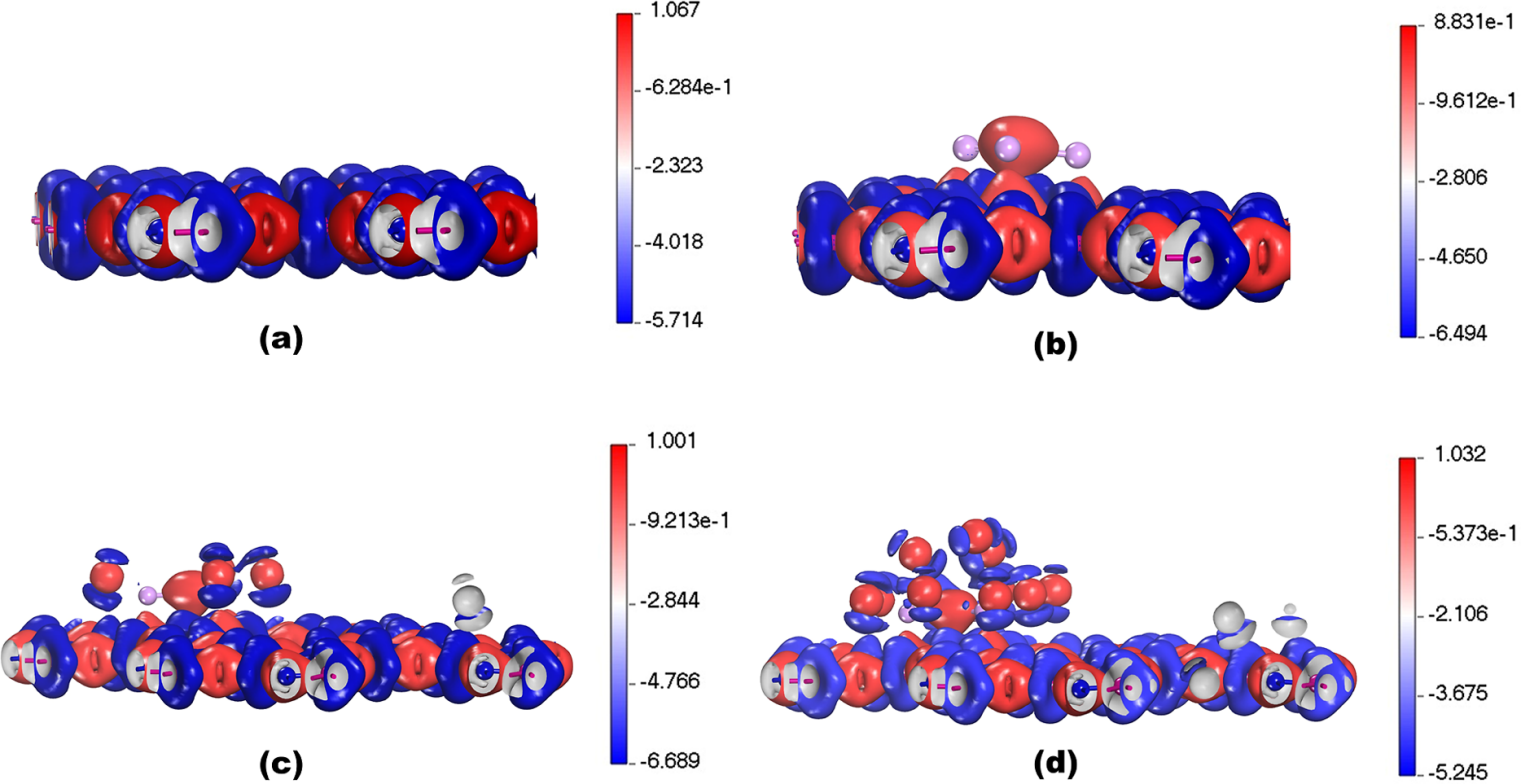
Deformation charge density for (a) IGP-SiC, (b) OLi_3_@IGP-SiC, (c) OLi_3_@IGP-SiC + 4H_2_, and (d) OLi_3_@IGP-SiC + 12H_2_ at 0.03 e/Bohr.^3^

Further, charge redistribution of different atoms of OLi_3_@IGP-SiC and OLi_3_@IGP-SiC + nH_2_ can also be investigated from Hirshfeld charge analysis (Figure S5). Variation of very little Hirshfeld electronic charge is noticed on the carbon (negative charge) and silicon (positive charge) atoms in the case of OLi_3_@IGP-SiC before and after the adsorption of hydrogen molecules. On the other hand, the highest electronic charge is gained by the oxygen atoms (−0.44 e.u. to −0.41 e.u.) due to their highest electronegativity. However, a significant change in the electronic charge has been found in the case of Li and H atoms. Charge on the Li atom has been estimated to be 0.39 e.u. in the case of OLi_3_@IGP-SiC, which decreases gradually and becomes 0.25 e.u. on adsorption of the maximum number of hydrogen molecules. Similarly, the positive charge on the hydrogen atom of adsorbed hydrogen molecules has been calculated to be 0.03 e.u. and decreases to 0.01 e.u. on reaching the saturation point.

To understand the orbital contribution in the adsorption of hydrogen molecules on OLi_3_@IGP-SiC, the PDOS analysis has been performed. The PDOS values of IGP-SiC, OLi_3_@IGP-SiC, OLi_3_@IGP-SiC + 4H_2_, and OLi_3_@IGP-SiC + 12H_2_ have been derived at the PBE/DNP level and are presented in Figure 6a–d. It is observed from Figure 6a–d that the C (2p) and Si (3p) orbitals undergo hybridization with other orbitals of the substrate across both valence and conduction bands. However, a marginal decrease in the peak height of the Si (3p) orbital is seen on the interaction with OLi_3_ (Figure 6b) as well as on adsorption of hydrogen molecules (Figure 6c,d), corresponding to smaller charge loss from pristine IGP-SiC. In contrast, a significant change in the orbital contribution of Li (2s) and H (1s) has been noticed. The contribution of the Li (2s) orbital is negligible in the valence band region but becomes more pronounced in the conduction band region. However, PDOS of Li (2s) orbital in OLi_3_@IGP-SiC is seen to be smaller in comparison to the isolated Li (2s) orbital in OLi_3_, corresponding to charge loss from the Li atom (Figure S5). Notably, both Li (2s) and H (1s) orbitals involve hybridization in the region of the valence band, as well as in the conduction band region, as visualized in Figure 6c,d. The contribution of the H (1s) orbital is more pronounced in comparison to the Li (2s) orbital in the valence bond region. Figure 6c, d shows more pronounced orbital contribution of H (1s) orbital of adsorbed H_2_ in the valence as well as in the conduction band regions in comparison to isolated H_2_ (Figure S6), and consequently, reduction of Li (2s) orbital is seen in the conduction band. This signifies the charge redistribution between H_2_ and OLi_3_@IGP-SiC, which enhances the polarization process in the adsorption of hydrogen.

**Figure 6:**
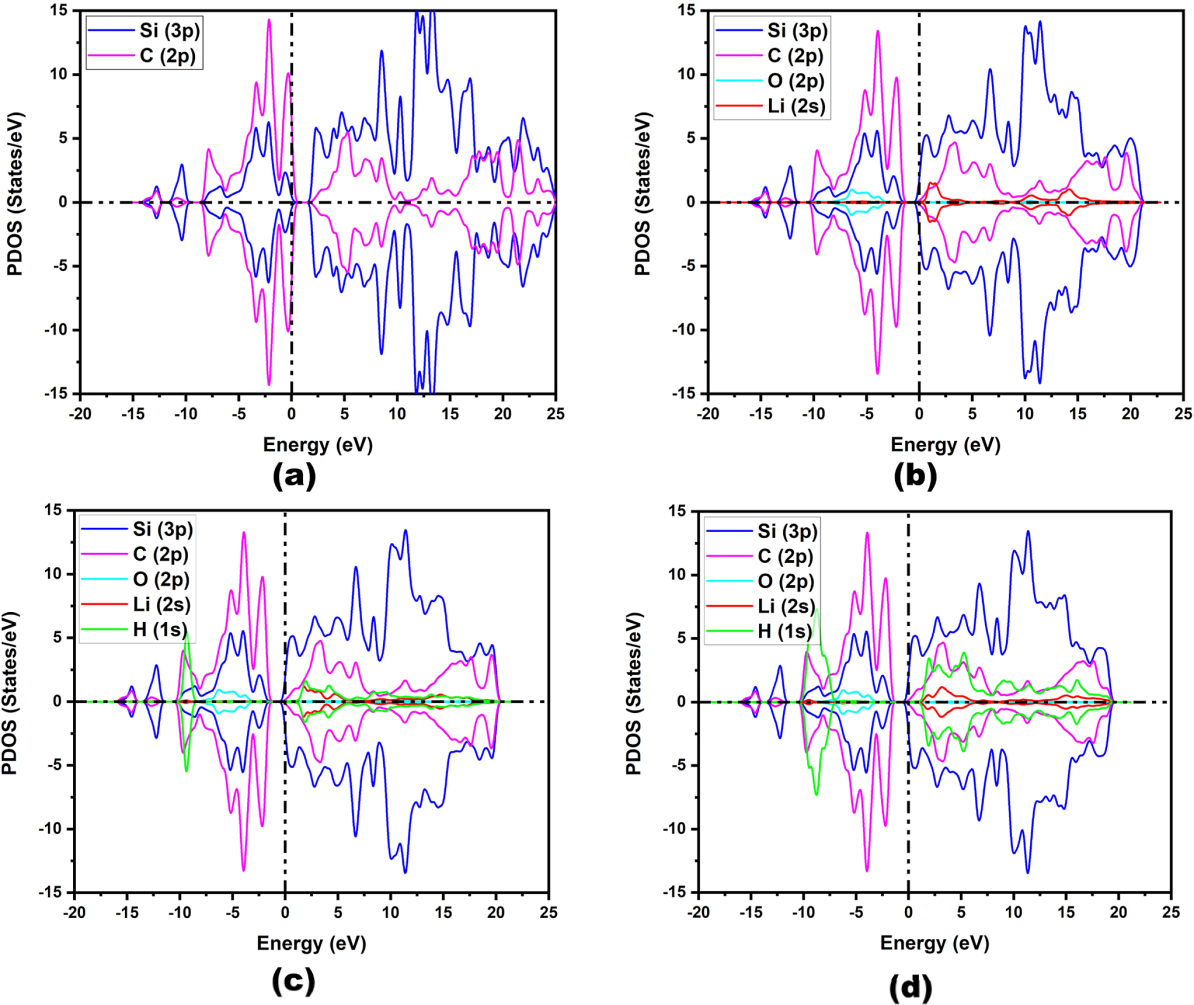
PDOS plot of (a) IGP-SiC, (b) OLi_3_@IGP-SiC, (c) OLi_3_@IGP-SiC + 4H_2_, and (d) OLi_3_@IGP-SiC +12H_2_.

Furthermore, to gain more insight into the depth of interaction of H_2_ with OLi_3_@IGP-SiC, reduced density gradient (RDG),^60^ a standard method to analyze the NCI, has been performed to observe the nature of interactions. RDG 3D iso-surface and 2D scatter plots for 6OLi_3_@IGP-SiC + 72H_2_ are presented in Figure 7. From the scatter plot in Figure 7a, the RDG vs sign (λ_2_)­ρ, the distribution of distinct regions can be seen as blue, green, and red, which indicates strong attraction, van der Waals interaction, and strong repulsion, respectively. The iso-surface of 6OLi_3_@IGP-SiC + 72H_2_ (Figure 7b) highlights the green regions between H_2_ and OLi_3_@IGP-SiC, signifying the van der Waals interaction between them. This analysis reveals that the interaction of hydrogen with OLi_3_@IGP-SiC occurs through polarization of charge and van der Waals interactions.

**Figure 7:**
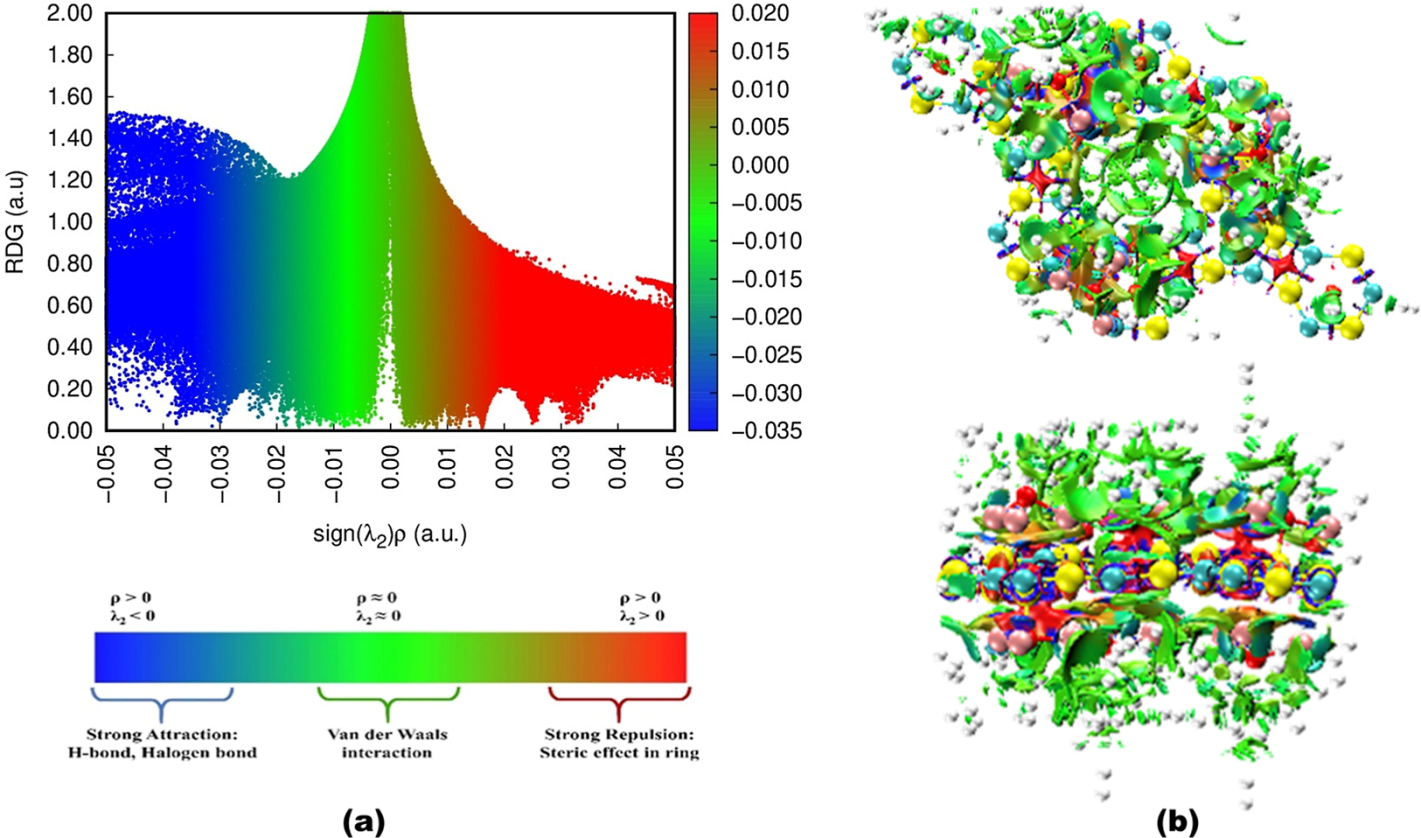
Noncovalent interaction (NCI) analysis of 6OLi_3_@IGP-SiC + 72H_2_. (a) Scatter plot of RDG vs sign (λ_2_)­ρ and (b) top and side view of RDG iso-surface.

### 3.4 Desorption Temperature

Hydrogen storage materials are expected to operate at ambient temperatures and pressures. According to DOE recommendations, the operating temperature and pressure have been set within the ranges of 233–358 K and 5–12 atm, respectively, for onboard hydrogen storage in light-duty vehicles. Desorption temperature (*T*_D_) is the temperature at which the desorption of adsorbed hydrogen occurs from the host surface. T_D_ is determined using eq 4, and the calculated values of *T*_D_ (at 1 atm pressure) are tabulated in Table 1. To facilitate the reversible binding of hydrogen, the storage material must be able to bind hydrogen molecules at elevated pressures and low temperatures.^61^ However, the material must be able to release hydrogen molecules at high temperatures for onboard storage applications. The minimum desorption temperature (Min *T*_D_) and maximum desorption temperature (Max *T*_D_) are calculated from the minimum and maximum*E*_H_2__^ad^ values. Similarly, the average desorption temperature (Avg *T*_D_) is evaluated from their average adsorption energy. The *E*_H_2__^ad^ values for minimum, maximum, and average hydrogen adsorptions are −0.14, −0.19, and −0.17 eV/H_2_, respectively. Min *T*_D_, Max *T*_D_, and Avg *T*_D_ of OLi_3_@IGP-SiC + *n*H_2_ (*n* = 1–12) are found to be 218, 296, and 265 K at 5 atm pressure, respectively, as shown in Figure 8. On the other hand, the observed values are 247, 335, and 299 K at 12 atm, respectively. As the pressure increases, the desorption temperature also increases, indicating that the desorption temperature varies with pressure. These desorption temperatures fall within the operating range, as recommended by the DOE.

**Figure 8:**
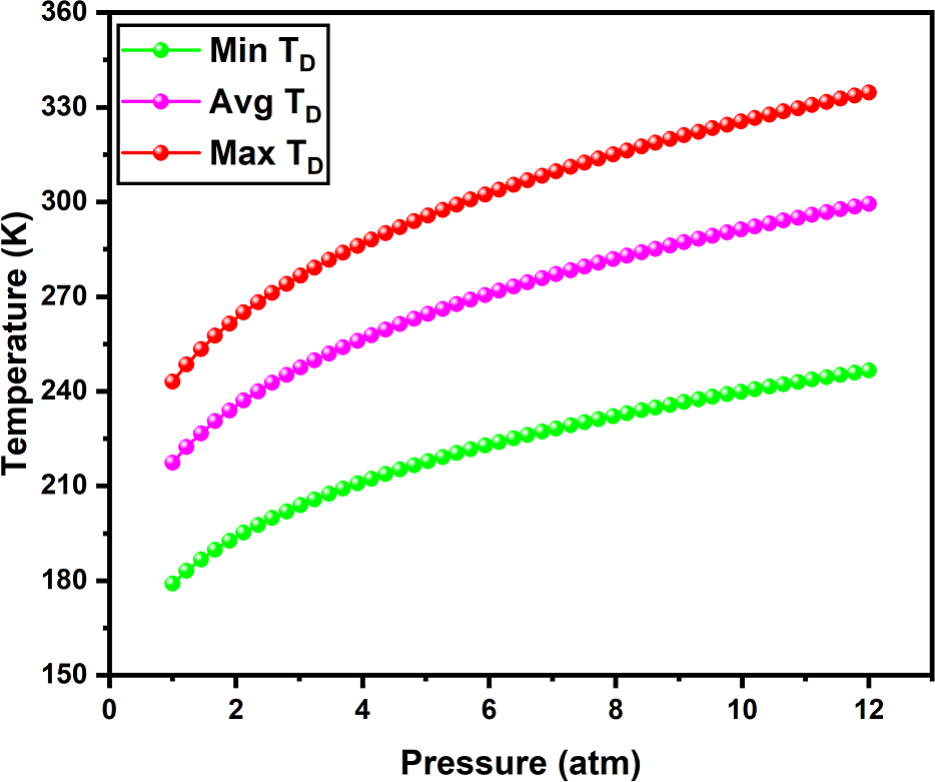
Variation of desorption temperature of OLi_3_@IGP-SiC + *n*H_2_ (*n* = 1–12) with pressure of 1–12 bar.

### 3.5 Hydrogen Occupation Number

To analyze the efficiency of hydrogen storage materials at different temperatures and pressures, the hydrogen occupation number (*N*) is calculated by using the mathematical formula mentioned in eq 5. The occupation number corresponds to the hydrogen adsorption–desorption reversible cycle exhibited by OLi_3_@IGP-SiC as a function of the temperature and pressure. Chemical potential (μH_2_) values with respect to *N* have been calculated using experimental values at temperatures ranging from 100 to 500 K and pressures 1 to 60 atm, by employing eq 6. Figure 9a illustrates the hydrogen occupation number as a function of pressure and temperature, which highlights the strong dependence of hydrogen uptake on both the pressure and temperature. To corroborate these results, the operational windows set by DOE are considered for onboard hydrogen storage in light-duty FCVs, which require delivery temperatures between −40 and 85 °C and a pressure range of 5–12 bar.^22^ As per the DOE delivery limits, the hydrogen uptake is calculated at constant pressures of 4.85 atm and is presented in Figure 9b, while at 11.97 atm, it is depicted in Figure 9c. The shaded region indicates the practical temperature window (233–358 K). In the interior limit of temperature 233 K, the highest hydrogen occupancy is 72H_2_ for both pressures. While the outer limit of temperature is 358 K, OLi_3_@IGP-SiC consistently adsorbs 45 and 49H_2_ molecules at 4.87 and 11.97 atm, respectively, corresponding to a hydrogen gravimetric storage capacity of approximately 7.12–7.70 wt %. Therefore, these findings demonstrate the potential of OLi_3_@IGP-SiC as a promising material for H_2_ storage.

**Figure 9:**
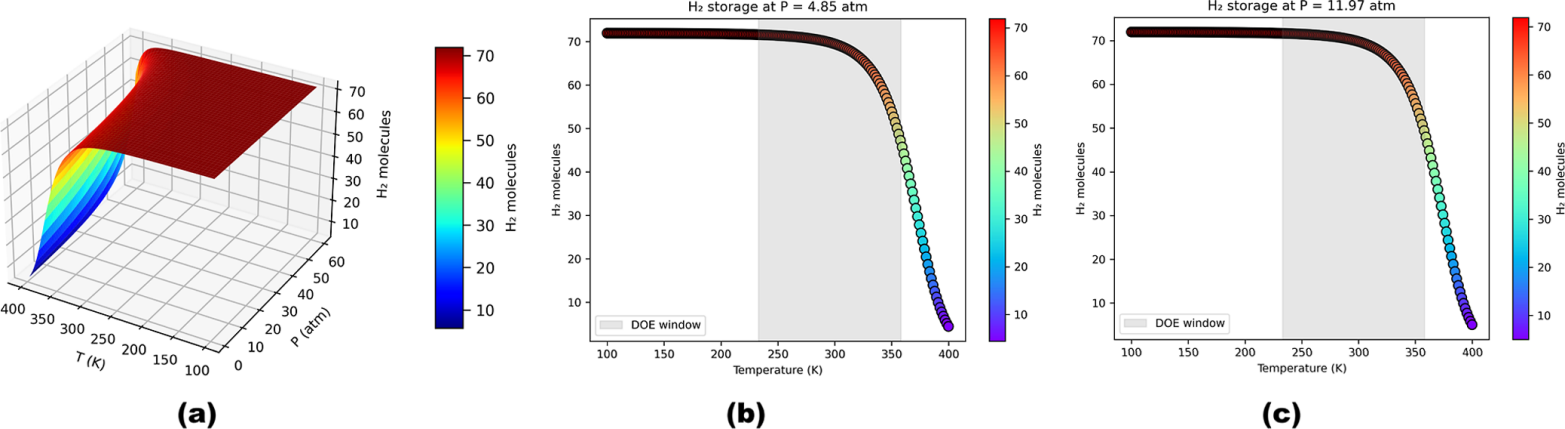
(a) Three-dimensional *N*–*P*–*T* surface plot showing the thermodynamic hydrogen uptake on OLi_3_@IGP-SiC as a function of temperature (*T*) and pressure (*P*). (b) Two-dimensional temperature-dependent hydrogen storage at *P* = 4.85 atm and (c) at *P* = 12.97 atm. The shaded region indicates the DOE temperature window (233–358 K).

### 3.6 Ab Initio Molecular Dynamics Simulations

To examine the reversibility of hydrogen storage, thermal stability of OLi_3_@IGP-SiC, and adsorption–desorption behavior of hydrogen molecules, an ab initio molecular dynamics (AIMD) simulation has been carried out for 5 ps simulation with a 1 fs time step at 100, 200, and 300 K. The visual representation of the final simulation of 6OLi_3_@IGP-SiC + 72H_2_ is shown in Figure 10. However, the fluctuations in the system’s energy and temperature are illustrated in Figure S5. It is observed that as the temperature increases, the desorption of hydrogen also gradually increases. At 100 K, the simulation illustrates that desorption of hydrogen initiates, and some of them stay far from OLi_3_@IGP-SiC, as shown in Figure 10a–c. Upon increasing the temperature to 200 K, desorption of hydrogen occurs over time (Figure 10d–f); however, the structural integrity remains stable as no structural deformation or metal clustering is observed. In contrast, in the 300 K simulation, all of the adsorbed hydrogens are completely desorbed and remain far away from 6OLi_3_@IGP-SiC, as shown in Figure 10g–i. At 300 K temperature, on complete desorption of hydrogen, 6OLi_3_@IGP-SiC is found to be thermally stable on the basis of the calculated minimum fluctuation of energy (Figures S7 and 10). According to the AIMD simulation, 6OLi_3_@IGP-SiC is found to be suitable for reversible hydrogen storage applications, as recommended by the DOE.

**Figure 10:**
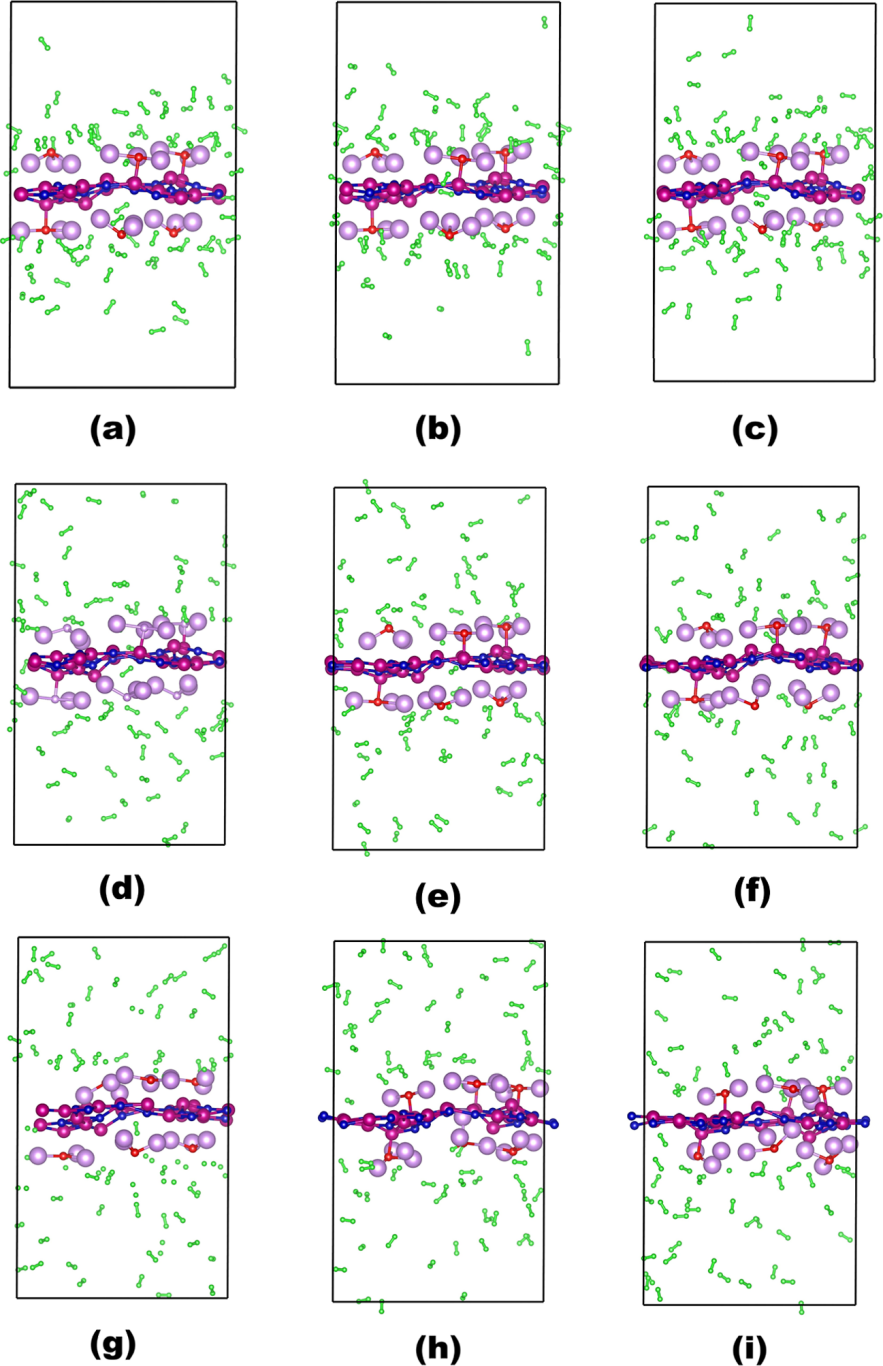
Snapshot of AIMD simulation for 6OLi_3_@IGP-SiC + 72H_2_ for (a–c) at 100 K; (d–f) at 200 K; and (g–i) at 300 K temperature.

### 3.7 Effect of the Electric Field in Hydrogen Storage

It is important to check the application of the external electric field along the perpendicular direction to the hydrogen adsorption on the IGP-SiC monolayer.^62–64^ The magnitude of the applied electric field strength is in the range of ±0.005 to ±0.055 V/Å. The outcome of one hydrogen molecule adsorption on OLi_3_@IGP-SiC (OLi_3_@IGP-SiC + 1H_2_) under the application of electric fields is shown in Figure 11. It is observed that with an increase in the positive electric field, the hydrogen adsorption energy gradually increases. At zero electric field, the hydrogen adsorption energy (*E*_H_2__^ad^) is −0.197 eV/H_2_. However, it increases to −0.657 eV/H_2_ when an electric field of magnitude +0.055 V/Å is applied. In contrast, upon the influence of negative electric field, hydrogen adsorption energy gradually decreases and then starts increasing. That is, the *E*_H_2__^ad^ is found to be −0.204 at an electric field of −0.055 V/Å. The Hirshfeld charges on Li and H at zero electric field are 0.36 e.u. and 0.028 e.u., respectively. When an electric field of +0.055 V/Å is applied, these values change to 0.32 e.u. for Li and 0.012 e.u. for H. This indicates a redistribution of electronic charge within the OLi_3_@IGP-SiC + 1H_2_ system. The enhancement in adsorption energy under the electric field is not primarily driven by increased charge transfer. Instead, this is attributed to the polarization of H_2_ molecules induced by the electric field, which strengthens the dipole-induced dipole interactions with the Li adsorption sites. Thus, the applied electric field significantly improves the hydrogen adsorption energy on OLi_3_@IGP-SiC.

**Figure 11:**
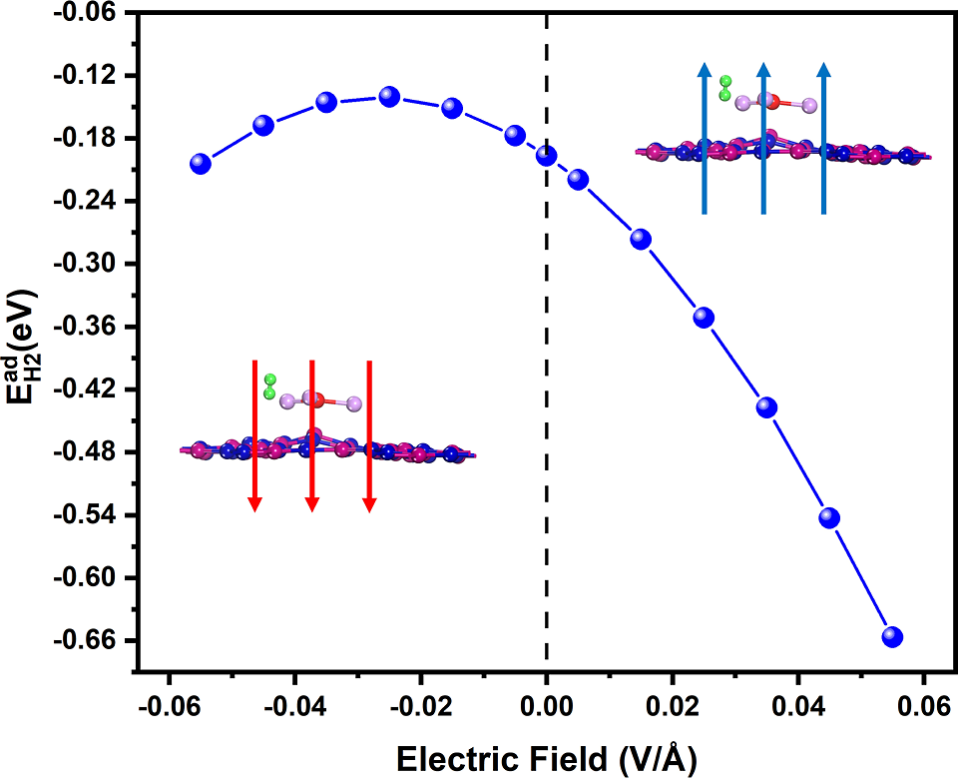
Effect of the electric field on hydrogen adsorption energy for OLi_3_@IGP-SiC + 1H_2_.

### 3.8 Hydrogen Diffusion Kinetics

Hydrogen diffusion energy barrier is essential for assessing the kinetic feasibility and reversibility of hydrogen storage. To investigate the hydrogen diffusion barrier, the climbing image-nudged elastic band (CI-NEB) method^65,66^ has been employed to examine the feasibility of hydrogen desorption kinetics. To compute the desorption barrier, two potential diffusion pathways have been selected, as shown in Figure 12a, and the investigated hydrogen diffusion profiles are illustrated in Figure 12b, in which ten images are used between the initial and final images. In pathway 1, the path length is 5.85 Å while the interimage distance is 0.65 Å. Along this path, the diffusion energy is calculated to be 0.131 eV. In contrast, pathway 2 has a length of 6.34 Å and an interimage distance of 0.70 Å, which demonstrates the diffusion energy barrier of 0.054 eV. This low diffusion energy barrier suggests the fast diffusion kinetics for reversible hydrogen storage.

**Figure 12:**
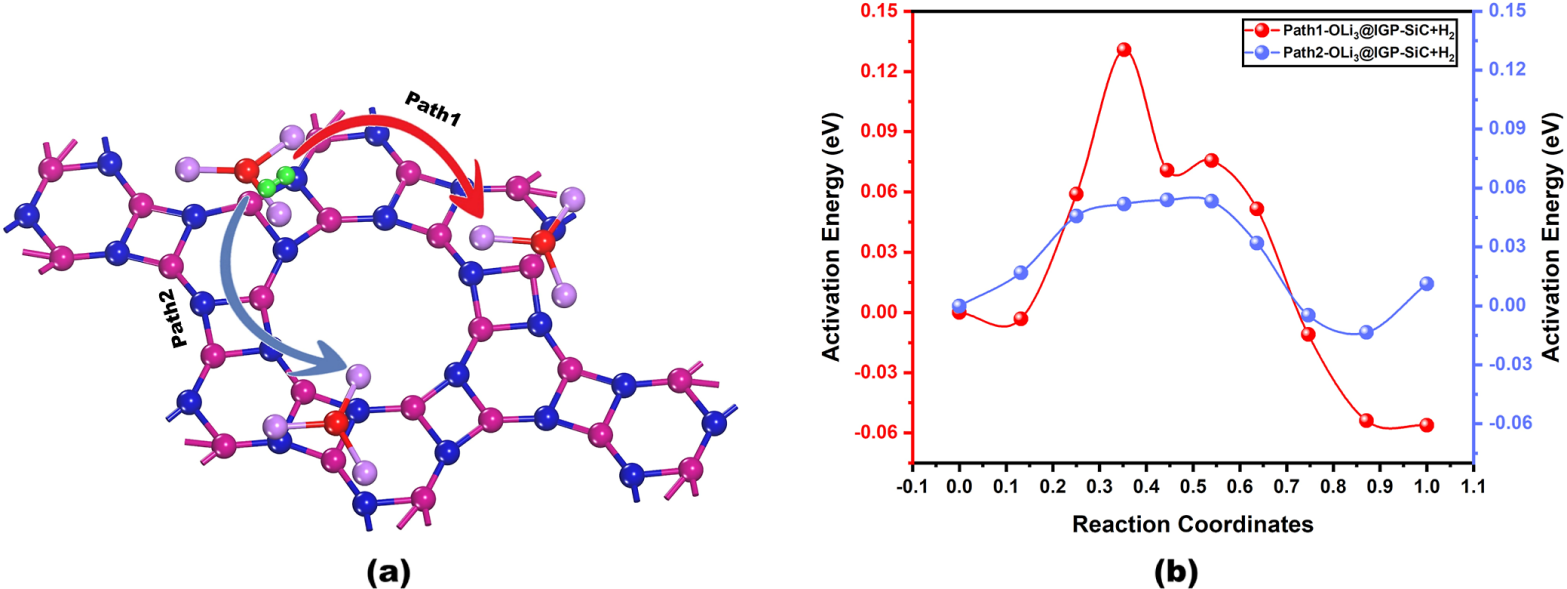
Hydrogen diffusion kinetics pathway for the OLi_3_@IGP-SiC system, (a) potential pathways, and (b) their diffusion energy barrier, employing the CI-NEB method.

## 4 Conclusions

In summary, we propose an IGP-SiC monolayer as a high-performance medium for hydrogen storage applications via superalkali OLi_3_ decoration. The binding energy of OLi_3_ on the IGP-SiC monolayer is evaluated, revealing that the most favorable binding (binding energy of −3.86 eV/OLi_3_) occurs when OLi_3_ is positioned near the top of the Si atom. Hirshfeld charge analysis confirmed the development of a positive charge on Li atoms of OLi_3_, which influences hydrogen adsorption. Each OLi_3_ cluster can adsorb up to 12H_2_ with an average adsorption energy of −0.17 eV/H_2_, aligning with the U.S. Department of Energy (DOE) recommendations for reversible hydrogen storage. OLi_3_@IGP-SiC can achieve a gravimetric storage density of 10.93 wt %, which exceeds the DOE target of 6.5 wt %. Mechanism of hydrogen adsorption on IGP-SiC is observed through orbital interaction, polarization of charge, and the van der Waals process. The thermal stability and reversibility of hydrogen storage are checked at 100, 200, and 300 K using the ab initio molecular dynamics (AIMD) simulation. The desorption temperature of 6OLi_3_@IGP-SiC + 72H_2_ is found to be 192 K using the van’t Hoff equation. On application of an electric field, the hydrogen adsorption energy on OLi_3_@IGP-SiC + 1H_2_ increases to −0.657 eV/H_2_ from −0.197 eV/H_2_. Moreover, the hydrogen diffusion barrier is found to be 0.054 eV, using the CI-NEB method. All of these outcomes concluded that the OLi_3_@IGP-SiC may be a promising hydrogen storage material for the transition to sustainable energy.

## Supplementary Material



## Notes

Relaxed structure of the pristine IGP-SiC unit cell and OLi_3_-decorated at different potential adsorption sites on IGP-SiC; band structure of pristine IGP-SiC and OLi_3_@IGP-SiC systems; Hirshfeld charge analysis of OLi_3_@IGP-SiC and their hydrogen adsorbed system; PDOS of isolated Li atom and H_2_; and AIMD output of temperature and energy fluctuations at 100, 200, and 300 K temperatures (PDF)
