# Supplementary material for: Exploring the Impact of Electric Field and OLi3 Decoration on Inorganic Graphenylene SiC for Reversible Hydrogen Storage: A First-Principles Investigation
Source: ACS Omega. 2026 Feb 2;11(6):10492–504. doi: 10.1021/acsomega.5c12031 (PMC12917855; doi:10.1021/acsomega.5c12031)
Supplement: Supplementary file 1 [file ao5c12031_si_001.pdf]

Supporting Information

Exploring the Impact of Electric Field and OLi_3 Decoration on Inorganic Graphenylene SiC for Reversible Hydrogen Storage: A First Principles Investigation

Amit Ramchiary[1], José A. S. Laranjeira[2]* and Paritosh Mondal[1]*

[1]Department of Chemistry, Assam University, Silchar-788011, Assam, India

[2]Modeling and Molecular Simulation Group, School of Sciences, São Paulo State University (UNESP), Bauru 17033-360 SP, Brazil

*Corresponding author: Email: jose.laranjeira@unesp.br, paritos_au@yahoo.co.in

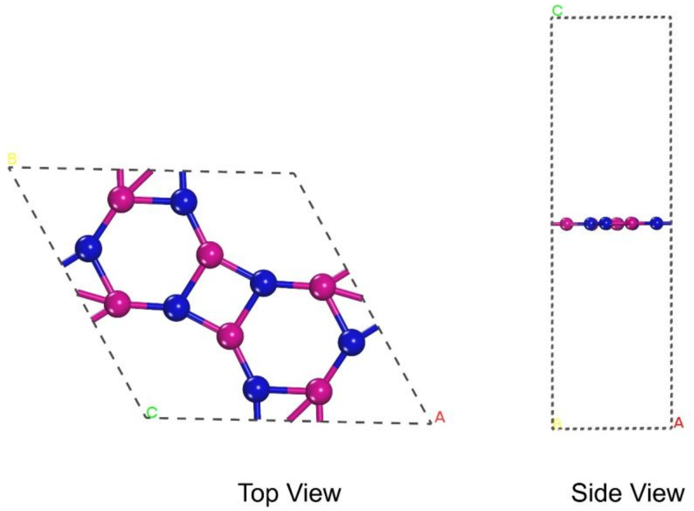

Figure S1. Relaxed geometry of top and side view pristine unit cell of IGP-SiC

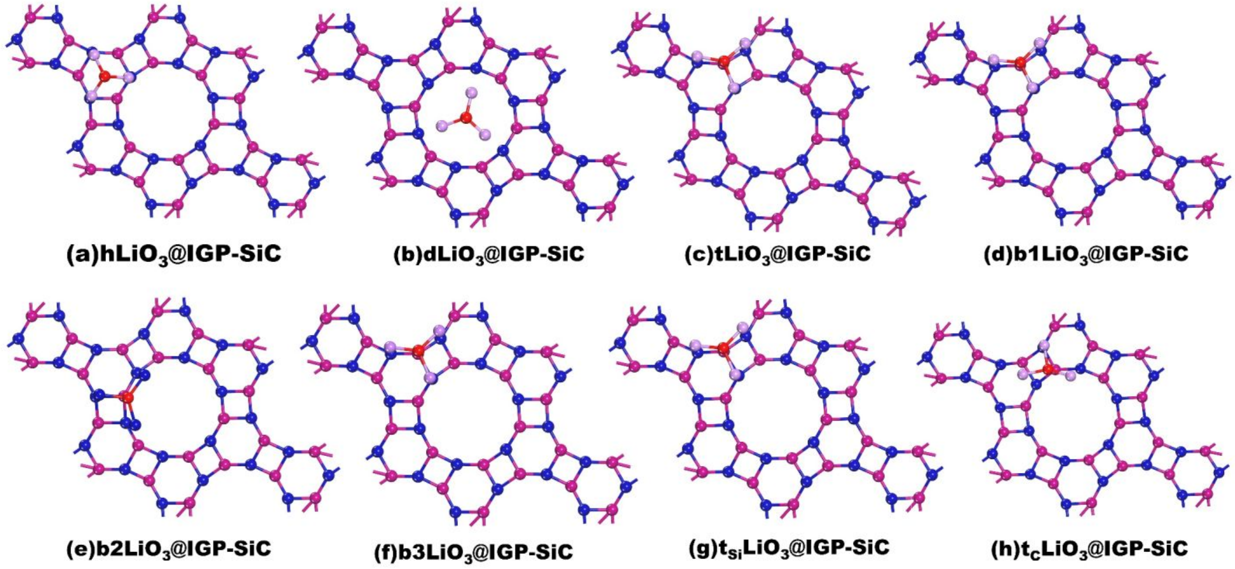

Figure S2. (a – h) Relaxed geometry of OLi_3 decorated IGP-SiC system at different possible adsorption sites.

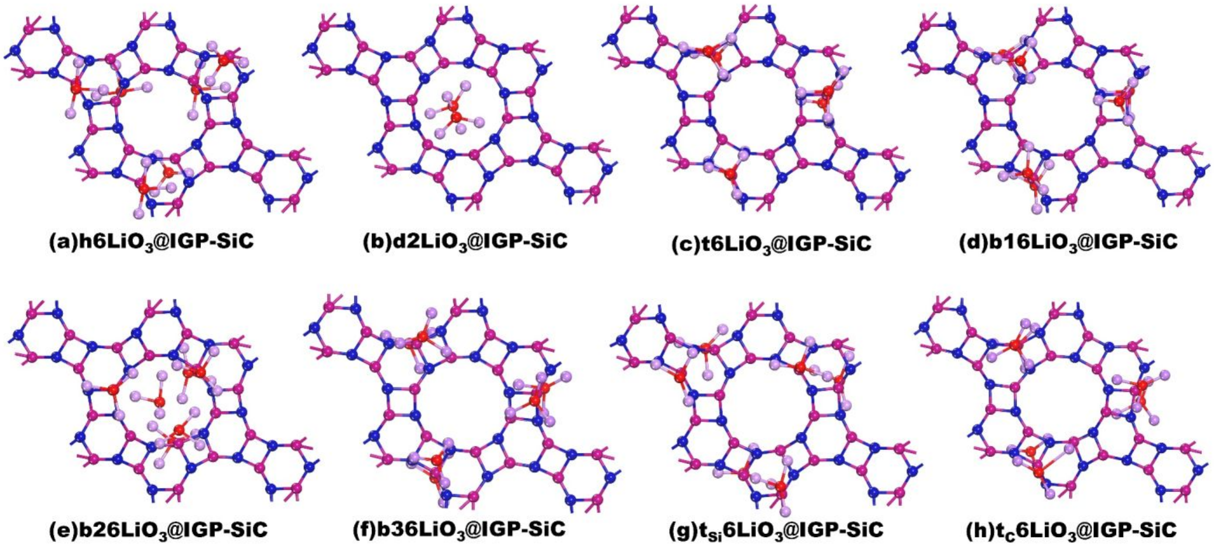

Figure S3. (a - h) Relaxed geometry of multiple OLi_3 decorated IGP-SiC system at different possible adsorption sites.

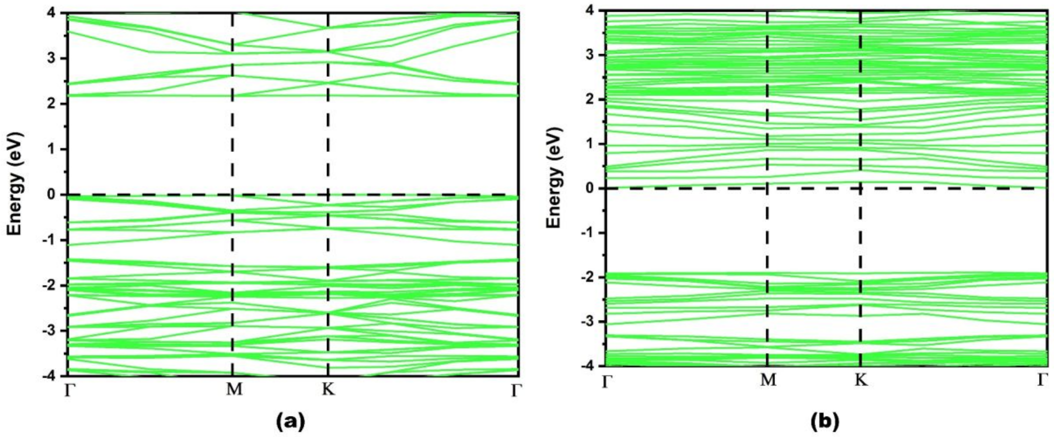

Figure S4. Electronic band structure of (a) pristine IGP-SiC and (b) OLi_3@IGP-SiC at GGA-PBE level

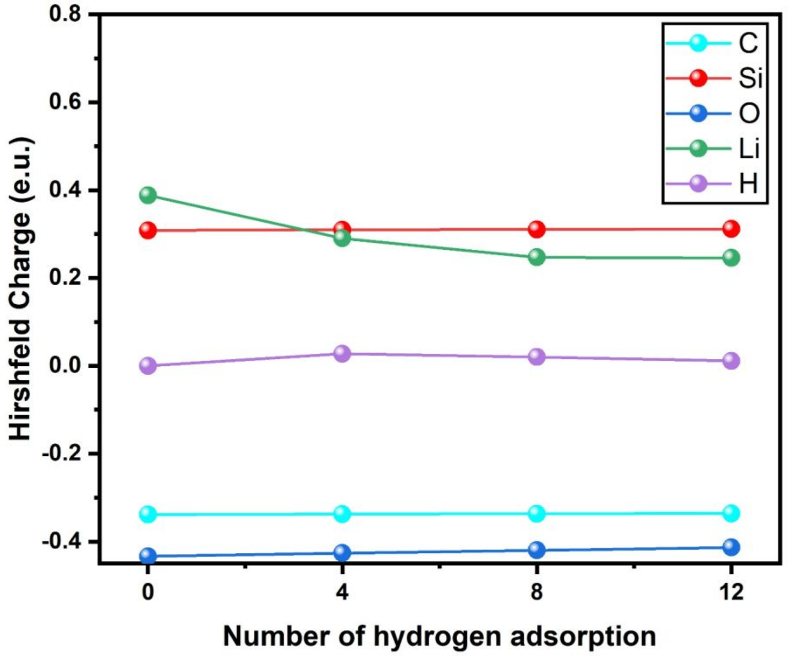

Figure S5. Hirshfeld charge analysis of OLi_3 decorated IGP-SiC (OLi_3@IGP-SiC) and hydrogen adsorption on OLi_3@IGP-SiC.

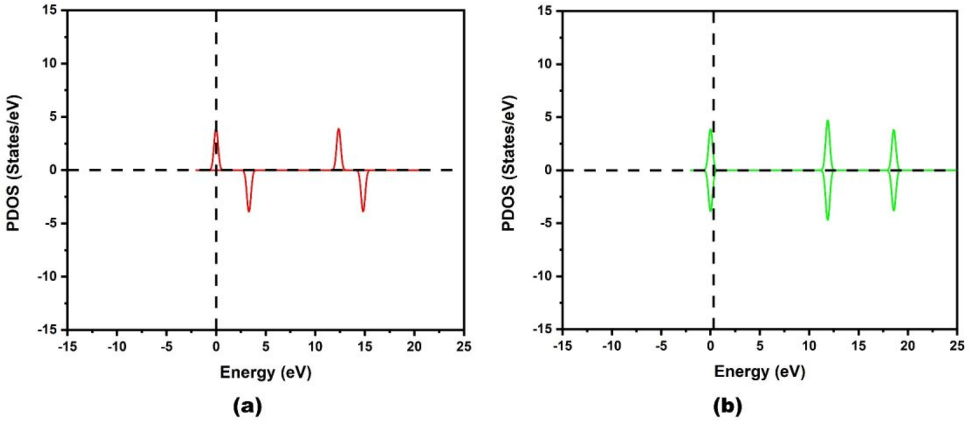

Figure S6. PDOS plot of (a) isolated Li atom, and (b) isolated H_2.

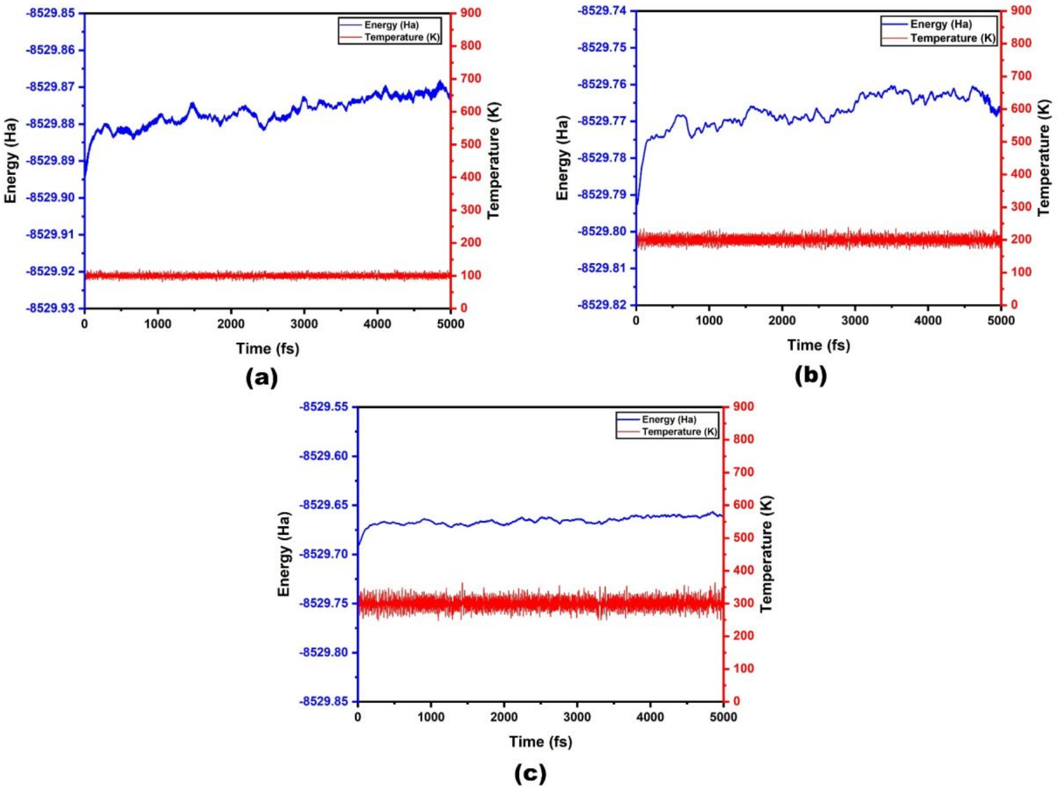

Figure S7. AIMD simulation outputs of $6OLi_3$@IGP-SiC + $72H_2$ during the 5ps simulation period; energy and temperature fluctuation at (a)100K, (b)200K, and (c)300K, respectively.
